# Unusual organ involvment in Henoch–Schönlein purpura patients; a roprt from Iran

**DOI:** 10.1186/1546-0096-9-S1-P99

**Published:** 2011-09-14

**Authors:** Vahid Ziaee, Mohamad-Hassan Moradinejad

**Affiliations:** 1 Department of Pediatrics, Tehran University of Medical Sciences, Tehran, IR Iran; 2 Growth and Development Research Center, Tehran University of Medical Sciences, Tehran, IR Iran

## Background

Henoch–Schönlein purpura (HSP) is the most common vasculitis in children. The common presentation in this disease is coetaneous, gastrointestinal (GI), joint and renal involvement, but sometimes unusual organ can be involvement in this disease.

## Aim

To evaluate unusual organ involvement in HSP.

## Methods

In a prospective study (2009 and 2010), all patients who had HSP diagnosis enrolled in this study. The diagnosis was established based on the ACR criteria. All organ involvement was evaluated and skin biopsy was done for all patients. In addition usual laboratory evaluation, other necessary investigation such as testicular Doppler ultrasound, brain CT scan was performed in selected cases.

## Findings

Out of 85 patients 34.2% was female and 65.8% were male (mean age 6.5± 3.9 years). Clinical symptoms were skin involvement in all patients (100%), GI involvement in 70.6%, joint and renal involvement (in acute phase) in 57.6% and 21.2%, respectively. Out of 56 male patients, 35.7% had genitalia involvement (75% scrotal swelling, 45% epididimo-orchitis like symptoms, 5% penis inflammation, and 5% meatus inflammation ).

Central nervous system (CNS) involvement was found in 8.2% (71.4% headache, seizures and sleep disorders each one 28.5%%, and confusion in one patient). Pinna perichondritis (PP) was detected in 3 patients (3.5%) which all of them were male and they had genitalia involvement. There is a significant relation between age and PP and all patients had age less than 5 years. There is no significant relation between age or sex and genitalia or CNS involvement.

## Conclusion

Genitalia involvement is a common in HSP. PP is a benign and uncommon involvement in HSP especially in male patients. Also, CNS involvement is uncommon but serious involvement is rare.

